# Factors That Affect Transfer of the IncI1 β-Lactam Resistance Plasmid pESBL-283 between *E*. *coli* Strains

**DOI:** 10.1371/journal.pone.0123039

**Published:** 2015-04-01

**Authors:** Nadine Händel, Sarah Otte, Martijs Jonker, Stanley Brul, Benno H. ter Kuile

**Affiliations:** 1 Dept. of Molecular Biology & Microbial Food Safety, University of Amsterdam, Swammerdam Institute of Life Sciences, Amsterdam, The Netherlands; 2 MicroArray Department and Integrative Bioinformatics Unit, Swammerdam Institute for Life Sciences, University of Amsterdam, Amsterdam, The Netherlands; 3 Office for Risk Assessment and Research, Netherlands Food and Consumer Product Safety Authority, Utrecht, The Netherlands; University Medical Center Utrecht, NETHERLANDS

## Abstract

The spread of antibiotic resistant bacteria worldwide presents a major health threat to human health care that results in therapy failure and increasing costs. The transfer of resistance conferring plasmids by conjugation is a major route by which resistance genes disseminate at the intra- and interspecies level. High similarities between resistance genes identified in foodborne and hospital-acquired pathogens suggest transmission of resistance conferring and transferrable mobile elements through the food chain, either as part of intact strains, or through transfer of plasmids from foodborne to human strains. To study the factors that affect the rate of plasmid transfer, the transmission of an extended-spectrum β-lactamase (ESBL) plasmid from a foodborne *Escherichia coli *strain to the β-lactam sensitive *E*. *coli* MG1655 strain was documented as a function of simulated environmental factors. The foodborne *E*. *coli *isolate used as donor carried a CTX-M-1 harboring IncI1 plasmid that confers resistance to β-lactam antibiotics. Cell density, energy availability and growth rate were identified as factors that affect plasmid transfer efficiency. Transfer rates were highest in the absence of the antibiotic, with almost every acceptor cell picking up the plasmid. Raising the antibiotic concentrations above the minimum inhibitory concentration (MIC) resulted in reduced transfer rates, but also selected for the plasmid carrying donor and recombinant strains. Based on the mutational pattern of transconjugant cells, a common mechanism is proposed which compensates for fitness costs due to plasmid carriage by reducing other cell functions. Reducing potential fitness costs due to maintenance and expression of the plasmid could contribute to persistence of resistance genes in the environment even without antibiotic pressure. Taken together, the results identify factors that drive the spread and persistence of resistance conferring plasmids in natural isolates and shows how these can contribute to transmission of resistance genes through the food chain.

## Introduction

Bacteria can become resistant against antibiotics by phenotypic adaptation, genetic changes or uptake and incorporation of resistance genes. Resistance conferring plasmids (R-plasmid) play an important role in the dissemination of antimicrobial resistance genes enabling the latter mechanism [1–3]. Transfer of R-plasmids through conjugation dramatically enhances the spread of antibiotic resistance. This in turn causes a range of problems, such as increased treatment costs and a lack of effective components against multidrug resistant pathogenic bacteria [2]. Molecular comparison between resistant *E*. *coli* isolates from poultry and humans revealed a high proportion of identical plasmid sequences [4], suggesting transmission of extended-spectrum β-lactamase (ESBL) genes through the food chain. Transfer of resistance genes from farms to humans is believed to be a result of direct contacts with animals or the consumption of affected food [5]. For instance, livestock-associated methicillin-resistant *Staphylococcus aureus* was shown to be transferred directly from animals to farm workers or close relatives [6,7]. Thus, transfer of R-plasmids from agricultural bacteria to human pathogens creates a potential link between selection for resistance at farms and clinical cases involving resistant strains.

Antibiotics belonging to the β-lactam class are among the most important antimicrobials used in veterinary and human medicine. Resistance to β-lactams is caused by overproduction of efflux pumps, resistance conferring mutations in penicillin binding proteins (PBPs) or due to the production of antibiotic inactivating enzymes, named β-lactamases [8]. So far, several hundreds of β-lactamases have been reported (http://www.lahey.org/studies) in many different genera of *Enterobacteriaceae* and *Pseudomonas aeruginosa* and new β-lactamases continue to emerge worldwide [9]. The spread of ESBL enzymes that are also able to hydrolyze cephalosporins, such as ceftazidime and cefotaxime [10,11] contributes to the overall increase in resistance. Plasmid-mediated ESBLs most commonly belong to the TEM and SHV gene families often found among *Enterobacteriaceae* [12,13], but since their discovery in 1986 a dramatic increase in CTX-M enzymes, which exhibit a higher β-lactamase activity against cefotaxime, has been reported [14,15]. At least 161 CTX-M variants have been identified (http://www.lahey.org/studies), whereby CTX-M-15 and CTX-M-14 are the most dominant enzymes [16].

Spreading of resistance genes involves two steps: the successful incorporation of the gene and the subsequent selection for resistance. This study focuses on the first step, as less information about it is available. Although the worldwide distribution of various ESBLs and a large variety of plasmids that encode for β-lactamases is reported, factors that drive the transfer of naturally occurring R-plasmids have rarely been described. Transfer of R-plasmids was observed in natural environments, such as seawater or in raw salmon and minced meat on a cutting board [17], even without any antibiotic pressure between bacterial species of different evolutionary or ecologically origins [17,18]. As transfer of resistance genes from the agricultural sector to human health care is a major hazard, we used an ESBL-carrying *E*. *coli* isolate from chicken meat as plasmid donor to study factors that drive the transfer of ESBL-plasmids. Analysis of the unknown plasmid revealed homology to a recently sequenced IncI1 plasmid carrying a β-lactamase of the CTX-M-1 type that has been identified in natural isolates of *E*. *coli* [19]. Cell density, availability of an energy source, growth rate and antibiotic pressure affected transfer rates between donor and acceptor cells. Furthermore, fitness costs of carrying the plasmid and compensatory mutations were studied to evaluate the possibility of persistence in the environment.

## Material and Methods

### Bacterial strains and antibiotics

Throughout this study a β-lactam sensitive, but chloramphenicol resistant (chlor^R^) *E*. *coli* MG1655 YFP (kindly provided by MB Elowitz) [20] was used as acceptor and an ESBL carrying *E*. *coli* isolated from chicken meat (ESBL242, kindly provided by B Wit of the Netherlands Food and Consumer Product Safety Authority) functioned as donor. Amoxicillin, ampicillin and chloramphenicol stock solutions (10 mg/ml) were 0.2-μm filter-sterilized and stored at 4°C. Filter-sterilized ceftazidime stock solutions (10 mg/ml) were stored at -20°C. Amoxicillin, ampicillin and chloramphenicol were purchased from Sigma-Aldrich (Steinheim, Germany) and ceftazidime from Fresenius Kabi (Schelle, Belgium).

### Plasmid transfer experiments using batch cultivations

Batch cultures of bacteria were grown at 37°C in a phosphate buffered (100 mM total NaH_2_PO_4_ and Na_2_HPO_4_, pH 6.9, Sigma-Aldrich, Steinheim, Deutschland) and defined minimal medium containing 55 mM glucose [21]. Media were autoclaved for 20 min at 120°C, with the exception of glucose (10 min at 110°C).

For plasmid transfer experiments in batch cultures, pre-cultures of acceptor and donor cells were grown separately in shake flasks overnight. To examine the effect of cell density and energy availability on transfer rates, cells were mixed 1:1 in defined minimal medium with 55mM glucose or without glucose. Since plasmid transfer occurred fast and at high rates, samples were taken after 1 hour of liquid mating and plated on antibiotic selective agar. To study the effect of varying antibiotic concentration on plasmid transfer, cells were mixed from overnight grown pre-cultures in a ratio of 1:1 to an initial OD_600_ (WPA S800 Spectrawave, De Meern, The Netherlands) of 0.01. Immediately after mixing, liquid mating was followed by culturing the cells at 37°C and 200 rpm for 24 hours. Samples were taken periodically to determine cell counts for acceptor, donor and transconjugant cells. The total numbers and ratio of acceptor, donor and transconjugant cells were determined by plating an appropriate dilution of the culture on antibiotic selective agar plates. For the selection of donor cells LB agar (5 g/l NaCl (Sigma-Aldrich, Steinheim, Germany), 2.5 g yeast extract (Scharlau-Microbiology, Barcelona, Spain), 5 g bacto-tryptone (Brunschwig chemie, Amsterdam, The Netherlands), Bacteriological agar (Scharlau-Microbiology, Barcelona, Spain)) plates containing 50 μg/ml amoxicillin or ampicillin were used. Acceptor cells were selected with 32 μg/ml chloramphenicol containing LB plates. The number of transconjugants that had evolved on chloramphenicol (32 μg/ml) and ampicillin or amoxicillin (50 μg/ml) containing plates were subtracted from the colony counts of donor and acceptor plates. The threshold for detection of transconjugant cells was 10 per mL. Mating of acceptor and donor cells on LB agar plates can be excluded as no transconjugants were observed when strains were cultured under conditions that did not allow plasmid transfer and subsequently plated on LB plates.

### Plasmid transfer experiments using continuous cultivations

In order to simulate conditions close to the natural environment with sub-optimal growth rates and a growth-limiting substrate, key experiments were repeated using continuous cultures. By adjusting the medium inflow and the waste outflow in the bioreactor and keeping growth conditions, such as temperature and pH, constant the growth rate of bacteria can be controlled. For continuous cultures a similar defined mineral medium as described for batch cultures was used. The glucose level was reduced to 5 mM and the Na_2_H_2_PO_4_ concentration to 10 mM. Continuous cultures were performed in a Sixfors (Infors AG, Bottmingen, Switzerland) fermenter system with 6 vessels having a working volume of 250 ml. Temperature and stirrer rate were kept constant at 37°C and 250 rpm, respectively. The pH was controlled by automatically pumping sterile 2 N NaOH (Sigma-Aldrich, Steinheim, Germany) into the vessel. After starting the cultures in batch modus for 24 hours, the culture was switched to continuous mode by activating the feed and waste pumps. Temperature, pH and stirrer rate were recorded by the controller in the Sixfors fermentation unit. Both acceptor and donor strains were grown separately without antibiotics, at the same growth rate. Growth conditions were maintained constant throughout experiments. When culture parameters, including OD and cell counts, remained constant for 5 to 7 volume changes, acceptor and donor cells were mixed in a ratio of 1:1 in a sterile empty vessel. The dilution rate (D), which at steady state equals μ, specific growth rate, of each individual and the subsequent mixed culture was identical. 5 mL of the culture was sampled periodically during 48 hours to follow OD, dry weight and cell count of each fraction (donor, acceptor and transconjugant) in the mixed culture. The total number and ratio of acceptor, donor and transconjugant cells was determined by plating an appropriate dilution on antibiotic selective agar as described earlier for batch cultivations.

### Measurement of maximum specific growth rate

Maximum specific growth rates of acceptor, donor and transconjugants were measured by inoculating overnight grown cells in 20 ml fresh defined minimal medium to an OD_600_ of 0.1 and following growth at 37°C and 200 rpm by measuring the OD600 (WPA S800 Spectrawave, De Meern, The Netherlands) every hour. Growth rates were calculated from data obtained in log phase and are reported as averages of two independent replicates. Student’s t-test was used to compare growth rates of acceptor and transconjugant cells. A p-value of ≤ 0.05 was considered significant.

### MIC measurement

The Minimum Inhibitory Concentration (MIC) values were measured by following growth in 96 well plates as described previously [22], using duplicate serial dilutions of a factor of 2, ranging from 1 to 1024 μg/ml of the antibiotic. Two additional wells were used to follow bacterial growth without any antibiotic. Cells were inoculated to an OD_600_ of 0.05 from an overnight in batch grown pre-culture. Growth in 96 well-plates was followed over time for 23h in a microtiter plate reader, measuring the optical density at 595 nm every 10 min, with shaking in between. The MIC was defined as the lowest concentration of antibiotic that reduced the growth to an OD_595_ of 0.2 or less after 23 hours. A Thermo Scientific Multiskan FC with SkanIt software was used for recording and analyzing the 96-well measurements.

### Measurement of β-lactamase activity

The β-lactamase activity was determined by using the chromogenic substrate nitrocefin (Merck, Darmstadt, Deutschland) according to an adapted protocol of O’Callaghan and coworkers [23]. Cells of an overnight grown culture were inoculated in fresh media and grown to an OD_600_ of 1.0. After harvesting and washing 1 ml of the culture with 100 mM sodium phosphate buffer (pH 7.0), cells were lysed using the same buffer containing 1% Triton X-100 (Merck, Darmstadt, Germany). Cell extracts were clarified by centrifugation for 1 min at 12.000 rpm. β-lactamase activity was determined by measuring the rate of nitrocefin hydrolysis (final assay concentration 100 mM) at 390 nm at 30°C in 100 mM sodium phosphate buffer (pH 7.0) using the BMG Fluostar Optima plate reader. Enzyme activity was normalized to the protein concentration determined with the Thermo Scientific Pierce Micro BCA Protein Assay Kit (Thermo Scientific, Rockford, USA). Specific β-lactamase activities are presented as nanomoles of nitrocefin hydrolyzed per minute per milligram of protein.

### Isolation of genomic and plasmid DNA

Genomic DNA was isolated using the DNeasy Blood and Tissue kit (Qiagen, Hilden, Germany) according to the manufactures instructions for Gram-negative bacteria. 0.5 mL of an overnight grown culture was used as starter material. To avoid co-purification of RNA, 4 μl of RNAse (stock 180mg/ml, Sigma/Aldrich, Steinheim, Germany) was added to the sample after cell lysis. For plasmid isolation the Qiagen Plasmid Maxi Kit (Hilden, Germany) was used according to the manufacturer’s instructions. Separation of the plasmid extract on a 1% agarose gel yielded two bands with a size of approximately 1kb and >10 kb. Subsequently, DNA was isolated and purified from the gel separately for each band using the QIAquick Gel Extraction Kit (Hilden, Germany). The amount and quality of DNA was measured on the NanoDrop ND-1000 (Thermo Scientific) and verified on a 1% agarose gel.

### Next-generation sequencing

gDNA libraries were generated according to the manufacturers’ protocols using the Ion Xpres Plus gDNA Fragment Library Preparations (Life Technologies). Shearing of 100 ng gDNA was performed using the Covaris M220 Focused-ultrasonicator following the 200-bp protocol provided by Life Technologies. Bar-coded libraries were prepared using the Ion Plus fragment library kit (Life Technologies) and the Ion Xpress DNA bar coding kit (Life Technologies) according to the 200-base-read Ion Proton libraries instructions of the manufacturer. The size distribution and yield of the barcoded libraries were assessed using the 2200 Tapestation System with Agilent High Sensitivity D1000 ScreenTapes (Agilent Technologies). Sequencing templates were prepared using the Ion PI Template OT2 200 Kit v3 on an Ion OneTouch 2 system and enriched on an Ion OneTouch ES system (Life Technologies). Sequencing was performed on the Ion Proton system using the Ion PI Chip v2 and the Ion PI Sequencing 200 kit v3 (Life Technologies) according to the manufacturers’ protocols.

### Data analysis

The FASTQ files were subjected to a quality control procedure, using in-house software and fastqc (http://www.bioinformatics.babraham.ac.uk/projects/fastqc/). Sequencing reads that have passed the quality control procedure were mapped onto the *E*. *coli* reference genome (str. K-12 substr. MG1655, complete genome; NC_000913.3) using Tmap [24]. The plasmids were identified by *de novo* assembly of the reads using CLC Genomics Workbench (http://www.clcbio.com/). The 1kb plasmid sample contained only a small number of reads (<1%) that mapped on the reference genome, and all reads were used for de-novo assembly of the plasmid. The assembly resulted in several potential plasmid sequences. The reads from the 1kb plasmid sample were mapped back onto these potential sequences using Tmap, to determine which of these sequences was most likely present in the sample in high abundances. The >10kb plasmid sample contained a large number of reads (>1%) that mapped on the reference genome, and only those reads not mapping on the genome were used for *de novo* assembly of the plasmid using CLC Genomics Workbench. The assembly resulted in several potential plasmid sequences with interpretable BLAST hits in the NCBI databases [25]. The reads from the >10kb plasmid sample were mapped onto these BLAST hits to determine if these sequences were likely to be present in the sample in high abundances. In addition, the sequencing reads from the acceptor, donor and transconjugant samples were mapped onto the plasmid sequences to determine whether the plasmids were present in these samples. Finally, single nucleotide variants, insertions and deletions were identified in the experimental *E*. *coli* samples compared to the reference genome using the Genome Analysis Toolkit (GATK; [26]), with BWA replaced by Tmap.

## Results

### Identification of plasmids from ESBL242 strain

A naturally occurring *E*. *coli* strain was used throughout that has been isolated from chicken meat. This strain was resistant against penicillin-like antibiotics as demonstrated by high MICs for ampicillin and amoxicillin ≥ 1024 μg/ml (Table 1), but not against cephalosporins (MIC for cefazidime 16 μg/ml). Isolation and sequencing of the plasmids it contained revealed one small plasmid with a length of 1554 bp. This small element showed 99.7% identity with a previously identified mobile element pJJ1886-1 (Accession number CP006784) which does not confer antibiotic resistance according to ResFinder 1.4 [27,28]. The second plasmid was identified as pESBL-283 (Accession number CP008736) with a size of 110137 bp. This plasmid belongs to IncI1 family and harbors a CTX-M-1 β-lactamase and plasmid addiction systems [19]. The reads of the sequence runs of both plasmids are accessible at NCBI SRA database: SRX878242.

**Table 1:** Minimum inhibitory concentration (MIC), maximum growth rate (μ_max_) and β-lactamase activity of acceptor, donor and transconjugant cells.

	*E*. *coli* MG1655/YFP	ESBL242	Transconjugants
MIC for ampicillin [μg/ml]	2–4	>1024	>1024
MIC for chloramphenicol [μg/ml]	128–256	4	128–256
μ_max_ [h^-1^]	0.71 ± 0.01	0.82 ± 0.01	0.67 ± 0.01^a^
β-lactamase activity^b^	21.6 ± 2.9	724.9 ± 104.2	704 ± 226.8^c^

^a^ Average maximum growth rate of three transconjugants randomly chosen from three different transfer experiments. All individual transconjugants were grown in two replicates.

^b^ Specific activity is reported in nanomoles of nitrocefin hydrolyzed per minute per milligram of protein. The results are presented as the means and standard deviations of two independent measurements.

^c^ β-lactamase activity was averaged from transconjugants obtained in two individual experiments

### Transfer of the resistance conferring plasmid with varying cell densities and energy availability in batch cultures

Initially the energy dependence of plasmid transfer and the effects of cell density and energy availability were examined. Preliminary experiments showed that the transfer of the resistance conferring plasmid pESBL-283 from *E*. *coli* donor to acceptor cells occurred fast and with high transfer rates. Therefore, to study the effect of cell density on plasmid transfer rates, a short incubation time of 1 hour was chosen to sufficiently discriminate and evaluate the outcome on the one hand, but also to simulate a short mating time that can for example occur during meat handling or wastewater treatment.

**Fig 1:**
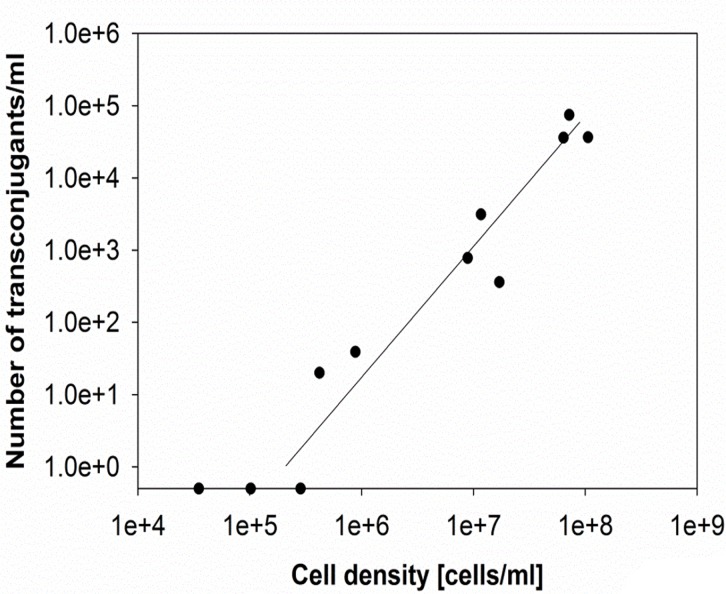
Number of evolved transconjugants/ml after co-culturing ESBL242 (donor, amp^R^) and *E*. *coli* MG1655 (acceptor, chlor^R^). Cells were mixed in a ratio of 1:1 at different total cell densities and incubated for 1 hour in batch culture.

In mineral medium without antibiotics, transfer from the ESBL242 donor (amp^R^, amox^R^) to the *E*. *coli* MG1655 acceptor (chlor^R^) occurred at high rates, but only at cell densities higher than 3 x 10^5^ cell/ml (Fig 1). No transfer was observed below a total cell density of 2.85 x 10^5^ cells/ml, when mixing donor and acceptor cells in a ratio of 1:1 and incubating for 1 hour. Increased cell densities resulted in higher transfer rates with a maximum of 7.46 x 10^4^ transconjugants/ml at a total cell density of 7.2 x 10^7^ cells/ml. In minimal medium that lacks glucose and antibiotics, no transconjugant cells were detected when donor and acceptor cells were mixed at a ratio of 1:1 in a range of 1 x 10^5^ cells/ml to 7.0 x 10^7^ cells/ml. This suggests that the transfer, incorporation and activation of the resistance conferring plasmid strongly depends on the availability of an energy source. Transconjugants obtained in three different experiments were tested regarding the resistance pattern and growth rate. All tested transconjugants showed a high MIC for chloramphenicol and ampicillin. The growth rate of transconjugant cells was 6% lower compared to that of the acceptor strain (Table 1). This difference was significant (p<0.05). The lower growth rate indicates that the high β-lactamase activity of cells carrying and expressing the ESBL-plasmid had a small but measurable impact on the fitness of the host.

### Transfer of the resistance conferring plasmid with varying antibiotic concentrations in batch cultures

**Fig 2:**
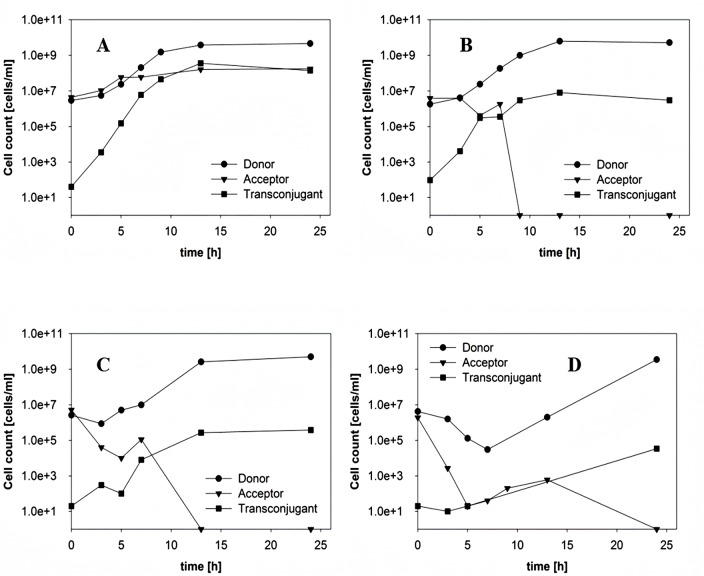
Transfer of pESBL-283 from ESBL242 (donor, amp^R^) to *E*. *coli* MG1655 (acceptor, chlor^R^) as a function of time during co-culture in a ratio of 1:1 with (A) 0 μg/ml, (B) 4 μg/ml, (C) 32 μg/ml and (D) 1024 μg/ml ampicillin in batch culture.

**Fig 3:**
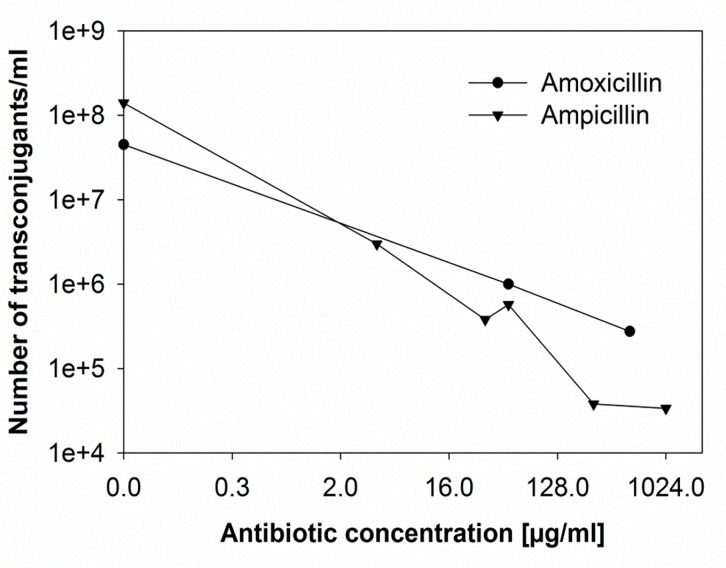
Number of evolved transconjugants/ml after co-culturing ESBL242 (donor, amp^R^) and *E*. *coli* MG1655 (acceptor, chlor^R^) for 24 hours in batch culture. Cells were mixed in a ratio of 1:1 with varying antibiotic concentrations.

To study the effect of antibiotic concentration on plasmid transfer, acceptor and donor cells were mixed in a ratio of 1:1 and growth of each fraction and the emergence of transconjugants were followed over time by plating samples on antibiotic selective agar. The highest plasmid transfer rate after 24 hours was observed in the absence of any antibiotics (Fig 2A–2D). The number of transconjugants and transfer rates obtained in the presence of ampicillin and amoxicillin were very similar (Fig 3). To examine the ability of transconjugant cells to function as donor of the ESBL-plasmid, acceptor and transconjugant cells were mixed in a ratio of 1:1. A transfer efficiency of 84 out of 100 cells indicated that the vast majority of acceptor cells had picked up the ESBL-plasmid. It must be kept in mind that transconjugants that emerged early in the experiment may have contributed to the rise in number of transconjugants at the end of the experiment. Exposing the mixed culture of ESBL242 donor and *E*. *coli* acceptor cells to 4 μg/ml ampicillin, corresponding to the MIC of the acceptor strain, resulted in a decrease of transconjugants/ml of about 100 fold (Figs 2A and 3). The acceptor cell number increased slower during the first 4 hours of co-culturing with 4 μg/ml ampicillin than in the absence of antibiotics, contributing to the observed lower number of transconjugants. With amoxicillin concentrations of 50 and 512 μg/ml this effect becomes even more obvious, as can be seen in the strong decrease of evolved transconjugants (Figs 2C, 2D and 3).

**Fig 4:**
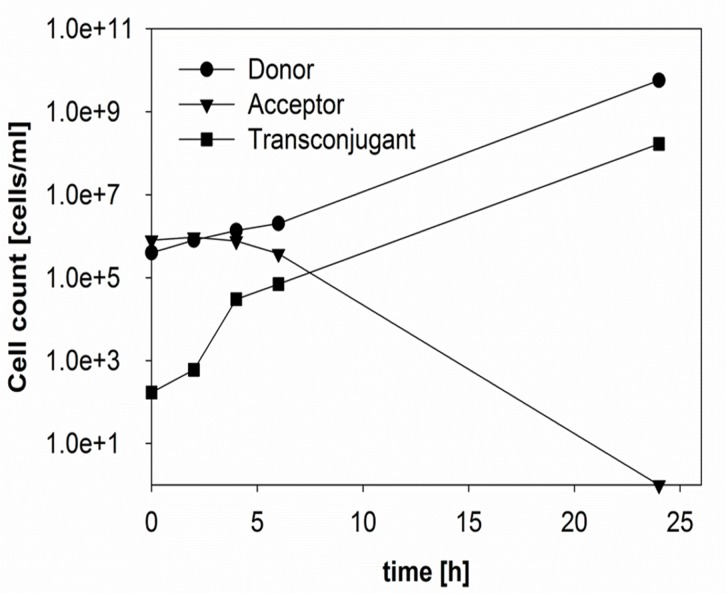
Transfer of pESBL-283 from ESBL242 (donor, amp^R^) to *E*. *coli* MG1655 (acceptor, chlor^R^) in a ratio of 1:1 with 8 μg/ml ampicillin and 4 μg/ml clavulanic acid.

Because of the global spread of ESBL carrying Enterobacteriaceae, β-lactam antibiotics are used often in combination with β-lactamase inhibitors, such as sulbactam or clavulanic acid, to restore antibiotic sensitivity. Generally, β-lactams and inhibitors are administered in a ratio of 2:1 [29]. Clavulanic acid itself had only little antimicrobial effect on the ESBL242 growth, but when grown with amoxicillin and clavulanic acid the MIC was reduced to 16 and 8 μg/ml, respectively. Co-incubation in the presence of half the MIC concentration, 8 μg/ml amoxicillin in combination with 4 μg/ml clavulanic acid immediately yielded transconjugants, and affected outgrowth of both strains during the first 6 hours (Fig 4). Based on the high β-lactam activity measured in donor cells, this period of time could correspond to the time needed for antibiotic degradation by non-inhibited β-lactamases. The number of transconjugants/ml determined after 24 h were similar to co-incubations in the absence of antibiotics (Figs 3 and 4).

In addition to penicillin-like antibiotics, transfer experiments were also carried out in the presence of the cephalosporin ceftazidime. Because of the low MICs of acceptor and donor cells with 1 μg/ml and 16 μg/ml, respectively, transfer experiments were only performed with 1 and 4 μg/ml ceftazidime. When exposed to 1 μg/ml ceftazidime, 120 transconjugant cells/ml were detected after 2 hours. However, prolonged exposure of cells to this concentration of ceftazidime led to eradication of both acceptors and transconjugants from the culture after 24 hours. In addition, growth of ESBL242 donor cells was hampered, as a high OD was only reached after 48 instead of 24 hours. In the presence of 4 μg/ml ceftazidime no plasmid transfer was observed at any time.

### Transfer of the resistance conferring plasmid with varying growth rate and antibiotic concentrations in continuous cultivations

**Fig 5:**
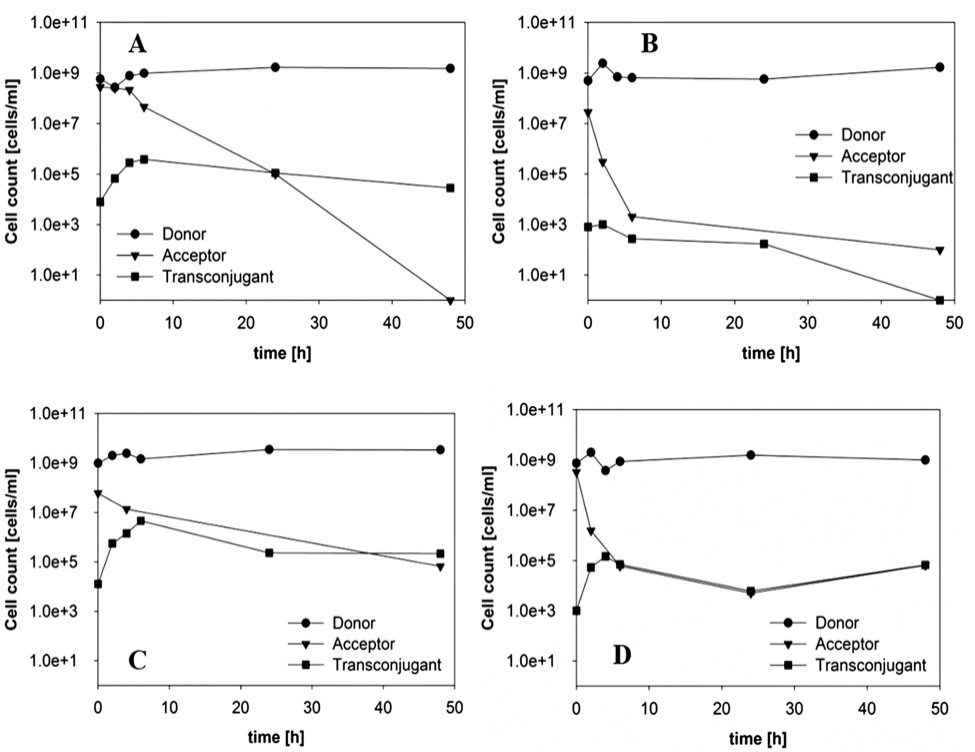
Transfer of pESBL-283 from ESBL242 (donor, amp^R^) to *E*. *coli* MG1655 (acceptor, chlor^R^). After donor and acceptor cells reached steady states in separate chemostats, cultures were mixed in a ratio of 1:1 in an empty reactor vessel and immediately supplied with fresh medium at the same dilution rate. Experimental conditions in each of the panels: (A) 0 μg/ml at D = 0.2 h^-1^, (B) 512 μg/ml ampicillin at D = 0.2 h^-1^, (C) 0 μg/ml at D = 0.4 h^-1^ and (D) 512 μg/ml ampicillin at D = 0.4 h^-1^.

**Fig 6:**
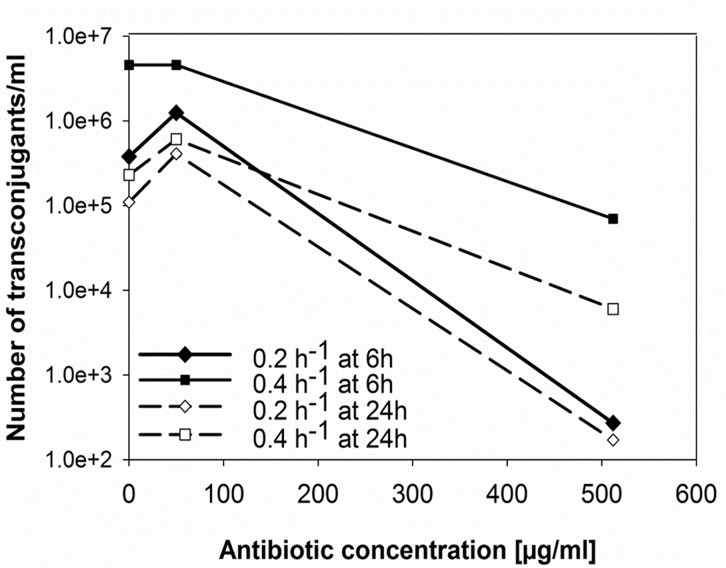
Number of transconjugants during continuous cultivation of ESBL242 (donor) and E. coli MG1655 (acceptor) cells at t = 6h or 24h. *E*. *coli* MG1655 and ESBL242 cells were cultivated separately and continuously at a dilution rate of D = 0.2 or 0.4 h^-1^ and subsequently mixed with a ratio of 1:1 (t = 0h).

During batch cultivations bacterial populations undergo all growth phases (lag, exponential and stationary phase) within 24 hours. Natural environments are often characterized by limitations of essential nutrients. Hence, growth at maximum rates and results obtained in batch cultures can deviate from outcomes obtained under conditions that are closer to natural circumstances. Therefore, key experiments were repeated using continuous cultivations that resemble a more natural environment with growth at reduced rates. Glucose was used as rate-limiting substrate to restrict growth and mimic a sub-maximal growth rate and limited energy levels. Simulations of the gastrointestinal tract commonly use dilution rates (D), which equals the growth rate μ in steady state, varying between 0.04 and 0.4 h^-1^ [30,31]. Therefore, transfer experiments were carried out in chemostats at D of 0.2 and 0.4 h^-1^, corresponding to approximately 0.3 and 0.6 times the maximum growth rate of *E*. *coli* in the minimal medium used respectively. As observed in batch cultivations, plasmid transfer rates decreased at high antibiotic concentrations (Fig 5). Increasing the growth rate resulted in a higher number of transconjugants/ml and thus higher transfer efficiencies (Fig 6). In agreement with results obtained in batch culture, ESBL-plasmids were picked up by almost every available acceptor cell (Fig 5). The observed higher biomass yield of the ESBL242 donor strain might have caused the reduction of acceptor and transconjugant cells over a long period of continuous growth (0.066 g_dry weight_ mm_glucose_^-1^ for acceptor versus 0.094 g_dry weight_ mm_glucose_^-1^ for donor cells at D = 0.2 h^-1^). Therefore the total number of transconjugants/ml after 6 hours seems higher compared to 24 hours (Figs 5 and 6).

### Genetic changes found in selected transconjugants

Transconjugants, evolved in either batch or continuous cultures were selected and sequenced in order to identify compensatory mutations in response to plasmid carriage (Tables 2 and 3). As to be expected, the resistance conferring plasmid pESBL-283 was recovered in the ESBL242 strain and all transconjugants. In contrast, the small plasmid with a size of 1554 bp was only found in some transconjugants independent of antibiotic use or culture condition (Table 2). This class of small cryptic plasmids was described earlier as selfish DNA that persists simply due to its ability to replicate and to its stability based on high copy number [32]

**Table 2:** Plasmids found in the acceptor, donor and selected transconjugants.

Sample	pJJ1886-1^a^	pESBL-283^b^
*E*. *coli* MG1655 (chlor^R^)	-	-
ESBL242 (amp^R^)	X	X
D = 0.4h^-1^ 0 μg/ml	-	X
D = 0.4h^-1^ 0 μg /ml	X	X
D = 0.4h^-1^ 50 μg /ml	-	X
D = 0.4h^-1^ 512 μg /ml	-	X
D = 0.4h^-1^ 512 μg /ml	X	X
Batch 0 μg /ml t = 0h	X	X
Batch 0 μg /ml t = 24h	-	X
Batch 50 μg /ml t = 24h	X	X
Batch 512 μg /ml t = 24h	X	X
Batch Amx/Clav t = 24h	-	X

^a^ Accession number CP006784

^b^ Accession number CP008736

Plasmids of ESBL242 were isolated, separated and identified by sequencing. The sequences can be accessed at NCBI SRA database: SRX878242. Transconjugants that evolved in different experiments were selected and sequenced.

**Table 3:** Mutations found in the genome of selected transconjugant cells.

Sample	Variant	Position	Gene	Function
D = 0.4h^-1^ 0 μg/ml	SNP	2 nucleotides downstream of *galS* C-> A	Not annotated	-
	SNP	AA 103 R—> C (CGT/TGT)	*rpoD*	RNA polymerase, sigma 70 (sigma D) factor
	SNP	AA 243 L-> Q (CTG/CAG)	*malT*	MalT transcriptional activator
	DEL	AA 81 CTG/C-G	*fliN*	flagellar motor switch protein FliN
D = 0.4h^-1^ 0 μg/ml	SNP	2 and 3 nucleotides downstream of *galS* GC-> AA	Not annotated	-
	SNP	AA 103 R—> C	*rpoD*	RNA polymerase, sigma 70 (sigma D) factor
	SNP	AA 243 L-> Q (CTG/CAG)	*malT*	MalT transcriptional activator
	DEL	AA 81 (CTG/C-G)	*fliN*	flagellar motor switch protein FliN
D = 0.4h^-1^ 50 μg/ml	SNP	23 nucleotides downstream of *yfjR*	Not annotated	-
	SNP	AA 77 V-> A (GTC/GCC)	*fimH*	minor fimbrial subunit, D-mannose specific adhesion
D = 0.4h^-1^ 512 μg/ml	SNP	AA 334 K-> I (AAA/ATA)	*rpoC*	RNA polymerase, β' subunit
D = 0.4h^-1^ 512 μg/ml	INS	36 nucleotides after AA 300	*rpoC*	RNA polymerase, β' subunit
Batch 0 μg/ml t = 0h	-	-	-	-
Batch 0 μg/ml t = 24h	SNP	AA 90 Q-> Stop (CAG/TAG)	*fliC*	flagellar biosynthesis; flagellin
	DEL	2 nucleotides downstream of *galS* C-> -	Not annotated	-
Batch 50 μg/ml t = 24h	DEL	15kb fragment from *flhA* to *otsA*		Fragment includes *flhDC* operon involved in flagellar biosynthesis and assembly
Batch 512 μg/ml t = 24h	SNP	AA 77 V-> A (GTC/GCC)	*fimH*	minor fimbrial subunit, D-mannose specific adhesin
	DEL	85 nucleotides downstream of *yfjM* (CGCACTATG/-)		CP4-57 prophage, antitoxin of the RnlA-RlnB toxin-antitoxin system
Batch Amx/Clav t = 24h	-	-	-	-

No consistent mutation was found in all transconjugants sequenced (Table 3). In total 11 different SNPs, one insertion and 4 deletions were found across all 10 transconjugants that have been analyzed. Two replicates from the same experiment showed similar, but not entirely identical outcomes. For example, one transconjugant evolved in continuous culture at D = 0.4 h^-1^ showed downstream of *galS* only one SNP, whereas the second transconjugant replicate colony had one additional SNP at the neighboring nucleotide. During continuous cultivation the highest number of at least four genetic alterations was found in the absence of antibiotic pressure. In transfer experiments with 512 μg/ml only one mutation was identified in two sequenced colonies in the β' subunit of the RNA polymerase *rpoC*. The transconjugant that has evolved in batch cultures directly after mixing the cells (t = 0h) in the absence of antibiotics showed no additional mutation, whereas the transconjugant from the same experiment but selected after 24 hours had a SNP in the flagellar biosynthesis protein FliC and a deletion 2 nucleotides downstream of *galS* that has also been identified in continuous cultures.

## Discussion

The rapid transfer of resistance genes in the absence of antibiotic pressure reported in this study partly explains the global spread of resistance conferring plasmids seen in the agricultural and medical sector [33,34]. Despite the fact that the probability of random cell-to-cell contact is higher in suspension, conjugation rates in suspension were similar to rates in biofilms [35]. In this study liquid mating was performed with a shaking speed of 200 rpm that can be considered as fast and could in theory shake donor and recipient apart. Nevertheless, the outcome of this study shows that transfer of the resistance conferring plasmid was not impaired by this rate of shaking. As found before [36], rates of transfer were highest in the absence of or with low antibiotic concentrations and increased at higher growth rates. Transfer rates observed in this study were much higher than measured for a plasmid (pDM0133, 95 kbp) that confers resistance to tetracycline which originated from a strain that has been isolated from broilers. [36]. Thus, conjugation rates of naturally occurring plasmids differ strongly. Transfer of resistance conferring elements depends on the donor cell and on information encoded on the plasmid itself [37]. Besides the CTX-M-1 β-lactamase, the plasmid used in this study carried genes for conjugational transfer proteins and plasmid addiction systems. Once the acceptor cell obtained pESBL-283, it replicates and maintains the resistance conferring element. We observed in our experiments that exposure to antibiotic was not needed to increase the fraction of plasmid carrying cells.

Energy availability and cell density seem to be important factors influencing the efficiency of transfer events. The latter one corresponds to the chance event of cell-to-cell contact and contact time that has been postulated to stimulate transfer events [35,38]. Conjugation as an energy demanding process requires the availability of an energy source as shown for *E*. *coli* (this study) and other bacteria [39]. However, in natural systems, such as seawater, nutrient limitation did not seriously impede conjugation, whereby starved cells were able to transfer plasmids as well [40]. The difference in methodology may explain varying outcomes. This study focused on short-term plasmid transfer events that can occur within one hour, for example during food processing or in the gastrointestinal tract. Studies using natural habitats that are nutrient limited consisted of experiments that measure transfer efficiencies over 3 days [40].

Plasmids are supposed to impose fitness costs on the host cell in the absence of the antibiotic [41]. Potential R-plasmid associated costs could be the consequence of plasmid regulation or expression of plasmid encoded genes [42]. Acquisition of the ESBL harboring plasmid caused a marginal but significant reduction of the maximum specific growth rate of 6% in transconjugants compared to recipient cells (Table 1). The presence of even small fitness costs is disadvantageous for cells constantly competing for nutrients in an antibiotic-free microbial community. Over evolutionary time fitness costs have decreased not only when resistance emerged *de novo* [43,44], but also when cells acquire resistance conferring plasmids by horizontal gene transfer [45–48]. Sequencing of selected transconjugants evolved in different experiments revealed no genetically identically mutation across all cells. However, genes involved in the biosynthesis and assembly of flagellar proteins seem to be hotspots for identified genetic alterations (Table 3). Without any antibiotic pressure mutations in flagellar proteins were identified only after 15,000 generations upon serial passaging of *E*. *coli* in minimal medium for overall 20,000 generations [49]. In agreement with this study, five population lineages of *E*. *coli* that were cultured in continuous logarithmic growth in glycerol minimal medium had mutations in the RNA polymerase or glycerol kinase, but no mutation was identified in motility associated genes [50]. In the present study, six out of ten sequenced transconjugants had mutations in genes involved in bacterial motility regardless of the antibiotic pressure. For instance, transconjugant cells evolved in batch culture sampled after 24 hours in the presence of 50 μg/ml ampicillin, corresponding to 6 generations, were characterized by the loss of the FlhD master operon that regulates the synthesis and assembly of flagella [51]. In addition, FlhD participates in the regulation of 29 operons, including genes involved in anaerobic metabolism and the Entner Douderroff pathway [52]. Loss of *flhD* allows *E*. *coli* to use a wider range of carbon sources compared to the wild-type [51]. Mutations that enhance the metabolic efficiency confer a colonization advantage as was observed for an *E*. *coli* MG1655 strain with a lack of *flhD* that evolved in a streptomycin-treated mouse intestine [53]. Motility is a phenotype that can be crucial to fitness in many environments entailing high energy demands [51,54]. Thus, alterations in motility associated genes can decrease the fitness burden and enhance metabolic efficiency. Based on evidence in the literature it can be assumed that the observed alterations in motility associated genes are linked to the event of plasmid transfer and serve as a strategy to reduce fitness costs. However, it cannot be excluded that the observed genetic alterations are caused by general adaptations to the experimental conditions.

Based on the observed high transfer rates without any antibiotic pressure and marginal fitness costs that can be compensated for, a picture emerges of well-adapted and easily spreading resistance plasmids. Microbial environments, such as the digestive tract, seem to be the optimal locus for conjugation by providing nutrients and the chance of cell-to-cell contact, even between different species [55,56]. Thus, this study illustrates the importance of limiting the use of antibiotics to its minimum to avoid selection for resistant strains, but also the need of adequate treatment and hygiene procedures to prevent transfer of resistance conferring plasmids to antibiotic sensitive cells.
